# 3-Phenyl-2-(1*H*-tetra­zol-1-yl)propanoic acid monohydrate

**DOI:** 10.1107/S1600536810038468

**Published:** 2010-09-30

**Authors:** Jie Xiao, Hong Zhao

**Affiliations:** a School of Chemistry and Chemical Engineering, Southeast University, Nanjing 210096, People’s Republic of China

## Abstract

In the title compound, C_10_H_10_N_4_O_2_·H_2_O, the dihedral angle between the tetra­zole and benzene rings is 63.24 (11)°. The crystal structure is stabilized by intra­molecular O—H⋯N and O—H⋯O hydrogen bonds.

## Related literature

For background to the applications of tetra­zole metal derivatives, see: Gaponik *et al.* (2006 ▶); Zhao *et al.* (2008 ▶); Xiao *et al.* (2009 ▶).
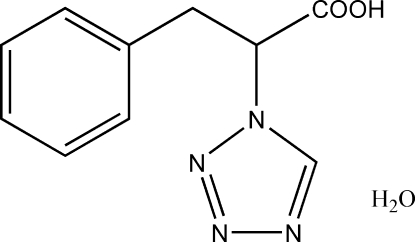


## Experimental

### 

#### Crystal data

C_10_H_10_N_4_O_2_·H_2_O
*M* *_r_* = 236.24
Orthorhombic, 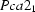

*a* = 24.001 (4) Å
*b* = 8.3769 (19) Å
*c* = 5.7455 (11) Å
*V* = 1155.1 (4) Å^3^
*Z* = 4
Mo *K*α radiation
μ = 0.10 mm^−1^
*T* = 293 K
0.40 × 0.25 × 0.10 mm

#### Data collection

Rigaku SCXmini diffractometer
Absorption correction: multi-scan (*CrystalClear*; Rigaku, 2005 ▶) *T*_min_ = 0.972, *T*_max_ = 0.987
11450 measured reflections
1461 independent reflections
1237 reflections with *I* > 2σ(*I*)
*R*_int_ = 0.049

#### Refinement

*R*[*F* ^2^ > 2σ(*F* ^2^)] = 0.045
*wR*(*F* ^2^) = 0.098
*S* = 1.11
1461 reflections
155 parameters
1 restraint
H-atom parameters constrained
Δρ_max_ = 0.15 e Å^−3^
Δρ_min_ = −0.17 e Å^−3^

### 

Data collection: *CrystalClear* (Rigaku, 2005 ▶); cell refinement: *CrystalClear*; data reduction: *CrystalClear*; program(s) used to solve structure: *SHELXS97* (Sheldrick, 2008 ▶); program(s) used to refine structure: *SHELXL97* (Sheldrick, 2008 ▶); molecular graphics: *SHELXTL/PC* (Sheldrick, 2008 ▶); software used to prepare material for publication: *SHELXTL/PC*.

## Supplementary Material

Crystal structure: contains datablocks I, global. DOI: 10.1107/S1600536810038468/rz2488sup1.cif

Structure factors: contains datablocks I. DOI: 10.1107/S1600536810038468/rz2488Isup2.hkl

Additional supplementary materials:  crystallographic information; 3D view; checkCIF report

## Figures and Tables

**Table 1:** Hydrogen-bond geometry (Å, °)

*D*—H⋯*A*	*D*—H	H⋯*A*	*D*⋯*A*	*D*—H⋯*A*
O2—H2⋯O1*W*^i^	0.82	1.74	2.552 (2)	174
O1*W*—H1*B*⋯N4^ii^	0.92	1.98	2.903 (4)	177
O1*W*—H1*A*⋯N3^iii^	0.89	2.12	3.003 (3)	171

Symmetry codes: (i) 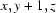; (ii) 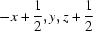; (iii) 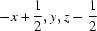.

**Fig. 1.**
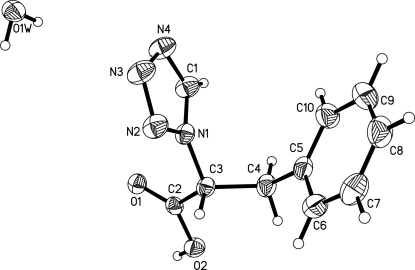
The molecular structure of the title compound, showing the atomic numbering scheme. The displacement ellipsoids are drawn at the 30% probability level.

**Fig. 2.**
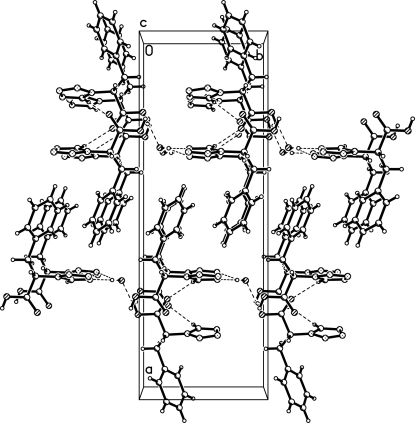
Packing diagram of the title compound, showing the structure down the c axis. Intermolecular hydrogen bonds are shown as dashed lines.

## supplementary crystallographic information

### Comment

Recently tetrazoles have been area of interest of coordination chemistry because of various applications of their metal derivatives (Gaponik *et al.*, 2006; Zhao *et al.*, 2008). A great variety of tetrazoles, especially substituted ones, are investigated as ligands. Recently, we have reported a few tetrazole compounds (Xiao *et al.*, 2009). As an extension of our work on the structural characterization of tetrazole compounds, the structure of the title compound is reported here.

In the molecule of the title compound (Fig. 1) bond lengths and angles have normal values. The dihedral angle between the planes of the tetrazole and phenyl rings is 63.24 (0.11)°. The crystal structure (Fig. 2) is stabilized by intramolecular O—H···N and O—H···O hydrogen bonds (Table 1).

### Experimental

2-Amino-3-phenylpropanoic acid (1.65 g, 10 mmol) and triethoxymethane (2.96 g, 20 mmol) was added to a mixture of sodium azide (0.65 g, 10 mmol) in acetic acid. After 3 h at 80°C, the mixture was cooled to room temperature and poured into 50 ml HCl (30%) to afford a white precipitate of the title compound. Colourless crystals suitable for X-ray diffraction were obtained after 3 days by slow evaporation of an ethanol solution.

### Refinement

All H atoms were detected in a difference map, but were placed in calculated positions and refined using a riding motion approximation, with C—H = 0.93–0.97 Å, O—H = 0.82–0.92 Å, and with *U*_iso_(H) = 1.2*U*_eq_(C) or 1.5*U*_eq_(O). In the absence of significant anomalous dispersion effects, Friedel pairs were merged.

### Crystal data

**Table:**

C_10_H_10_N_4_O_2_·H_2_O	*F*(000) = 496
*M**_r_* = 236.24	*D*_x_ = 1.358 Mg m^−^^3^
Orthorhombic, *P**c**a*2_1_	Mo *K*α radiation, λ = 0.71073 Å
Hall symbol: P 2c -2ac	Cell parameters from 2576 reflections
*a* = 24.001 (4) Å	θ = 2.4–27.5°
*b* = 8.3769 (19) Å	µ = 0.10 mm^−^^1^
*c* = 5.7455 (11) Å	*T* = 293 K
*V* = 1155.1 (4) Å^3^	Prism, colourless
*Z* = 4	0.40 × 0.25 × 0.10 mm

### Data collection

**Table:**

Rigaku SCXmini diffractometer	1461 independent reflections
Radiation source: fine-focus sealed tube	1237 reflections with *I* > 2σ(*I*)
graphite	*R*_int_ = 0.049
Detector resolution: 13.6612 pixels mm^-1^	θ_max_ = 27.5°, θ_min_ = 2.4°
ω scans	*h* = −30→31
Absorption correction: multi-scan (*CrystalClear*; Rigaku, 2005)	*k* = −10→10
*T*_min_ = 0.972, *T*_max_ = 0.987	*l* = −7→7
11450 measured reflections	

### Refinement

**Table:**

Refinement on *F*^2^	Primary atom site location: structure-invariant direct methods
Least-squares matrix: full	Secondary atom site location: difference Fourier map
*R*[*F*^2^ > 2σ(*F*^2^)] = 0.045	Hydrogen site location: inferred from neighbouring sites
*wR*(*F*^2^) = 0.098	H-atom parameters constrained
*S* = 1.11	*w* = 1/[σ^2^(*F*_o_^2^) + (0.045*P*)^2^ + 0.0812*P*] where *P* = (*F*_o_^2^ + 2*F*_c_^2^)/3
1461 reflections	(Δ/σ)_max_ < 0.001
155 parameters	Δρ_max_ = 0.15 e Å^−^^3^
1 restraint	Δρ_min_ = −0.17 e Å^−^^3^

### Special details

**Table:**

Geometry. All e.s.d.'s (except the e.s.d. in the dihedral angle between two l.s. planes) are estimated using the full covariance matrix. The cell e.s.d.'s are taken into account individually in the estimation of e.s.d.'s in distances, angles and torsion angles; correlations between e.s.d.'s in cell parameters are only used when they are defined by crystal symmetry. An approximate (isotropic) treatment of cell e.s.d.'s is used for estimating e.s.d.'s involving l.s. planes.
Refinement. Refinement of *F*^2^ against ALL reflections. The weighted *R*-factor *wR* and goodness of fit *S* are based on *F*^2^, conventional *R*-factors *R* are based on *F*, with *F* set to zero for negative *F*^2^. The threshold expression of *F*^2^ > σ(*F*^2^) is used only for calculating *R*-factors(gt) *etc*. and is not relevant to the choice of reflections for refinement. *R*-factors based on *F*^2^ are statistically about twice as large as those based on *F*, and *R*- factors based on ALL data will be even larger.

### Fractional atomic coordinates and isotropic or equivalent isotropic displacement parameters (Å^2^)

**Table:**

	*x*	*y*	*z*	*U*_iso_*/*U*_eq_	
C1	0.17744 (13)	0.5430 (3)	0.8029 (6)	0.0523 (7)	
H1	0.1849	0.5834	0.6553	0.063*	
C2	0.22253 (10)	0.8730 (3)	0.9938 (5)	0.0370 (5)	
C3	0.16456 (9)	0.8029 (3)	1.0194 (5)	0.0385 (6)	
H3	0.1523	0.8234	1.1793	0.046*	
C4	0.12188 (11)	0.8794 (3)	0.8571 (6)	0.0494 (7)	
H4A	0.1215	0.9937	0.8834	0.059*	
H4B	0.1332	0.8612	0.6972	0.059*	
C5	0.06378 (11)	0.8150 (3)	0.8910 (5)	0.0466 (6)	
C6	0.03288 (12)	0.8572 (4)	1.0852 (6)	0.0605 (8)	
H6	0.0480	0.9258	1.1957	0.073*	
C7	−0.02045 (13)	0.7972 (4)	1.1151 (7)	0.0738 (11)	
H7	−0.0410	0.8264	1.2455	0.089*	
C8	−0.04305 (13)	0.6958 (4)	0.9550 (8)	0.0714 (10)	
H8	−0.0787	0.6551	0.9767	0.086*	
C9	−0.01295 (13)	0.6545 (4)	0.7634 (8)	0.0728 (10)	
H9	−0.0282	0.5858	0.6535	0.087*	
C10	0.03997 (12)	0.7138 (4)	0.7312 (6)	0.0617 (8)	
H10	0.0600	0.6850	0.5991	0.074*	
N1	0.16699 (8)	0.6303 (2)	0.9891 (4)	0.0398 (5)	
N2	0.15902 (10)	0.5316 (3)	1.1690 (5)	0.0540 (7)	
N3	0.16442 (12)	0.3885 (3)	1.0871 (5)	0.0611 (7)	
N4	0.17577 (12)	0.3922 (3)	0.8561 (5)	0.0594 (7)	
O1	0.26404 (6)	0.79317 (19)	0.9755 (4)	0.0431 (4)	
O2	0.22055 (7)	1.02860 (19)	0.9997 (5)	0.0489 (4)	
H2	0.2522	1.0647	0.9909	0.073*	
O1W	0.31635 (8)	0.1603 (2)	0.9820 (4)	0.0574 (5)	
H1A	0.3191	0.2341	0.8710	0.086*	
H1B	0.3195	0.2313	1.1033	0.086*	

### Atomic displacement parameters (Å^2^)

**Table:**

	*U*^11^	*U*^22^	*U*^33^	*U*^12^	*U*^13^	*U*^23^
C1	0.0678 (18)	0.0421 (16)	0.0470 (17)	0.0014 (14)	0.0082 (15)	0.0055 (13)
C2	0.0443 (12)	0.0387 (12)	0.0281 (11)	−0.0003 (9)	−0.0035 (12)	0.0023 (13)
C3	0.0404 (12)	0.0343 (11)	0.0408 (15)	0.0011 (9)	0.0001 (11)	0.0066 (11)
C4	0.0468 (14)	0.0435 (14)	0.0579 (17)	0.0004 (11)	−0.0052 (13)	0.0097 (14)
C5	0.0420 (14)	0.0426 (14)	0.0553 (16)	0.0051 (11)	−0.0044 (13)	0.0077 (13)
C6	0.0555 (18)	0.0553 (17)	0.071 (2)	0.0057 (14)	0.0008 (17)	−0.0098 (16)
C7	0.058 (2)	0.081 (2)	0.082 (3)	0.0169 (17)	0.0193 (19)	−0.001 (2)
C8	0.0429 (15)	0.074 (2)	0.098 (3)	−0.0014 (14)	0.000 (2)	0.004 (2)
C9	0.055 (2)	0.078 (2)	0.086 (3)	−0.0076 (16)	−0.012 (2)	−0.012 (2)
C10	0.0507 (17)	0.072 (2)	0.0621 (19)	0.0008 (15)	−0.0013 (16)	−0.0097 (17)
N1	0.0407 (10)	0.0357 (10)	0.0429 (11)	−0.0008 (8)	0.0012 (11)	0.0081 (11)
N2	0.0709 (17)	0.0437 (14)	0.0473 (13)	0.0007 (12)	0.0061 (13)	0.0108 (12)
N3	0.0820 (18)	0.0393 (14)	0.0619 (16)	0.0022 (12)	0.0129 (15)	0.0076 (13)
N4	0.0760 (17)	0.0398 (14)	0.0624 (17)	0.0014 (12)	0.0127 (14)	0.0033 (13)
O1	0.0433 (10)	0.0469 (9)	0.0391 (9)	0.0032 (7)	0.0022 (9)	0.0037 (9)
O2	0.0489 (9)	0.0371 (9)	0.0607 (11)	−0.0051 (7)	−0.0016 (12)	−0.0002 (11)
O1W	0.0674 (12)	0.0484 (10)	0.0564 (11)	−0.0175 (8)	−0.0066 (13)	0.0071 (12)

### Geometric parameters (Å, °)

**Table:**

C1—N4	1.301 (4)	C6—H6	0.9300
C1—N1	1.320 (4)	C7—C8	1.365 (5)
C1—H1	0.9300	C7—H7	0.9300
C2—O1	1.205 (3)	C8—C9	1.362 (6)
C2—O2	1.305 (3)	C8—H8	0.9300
C2—C3	1.517 (3)	C9—C10	1.376 (4)
C3—N1	1.457 (3)	C9—H9	0.9300
C3—C4	1.526 (4)	C10—H10	0.9300
C3—H3	0.9800	N1—N2	1.337 (3)
C4—C5	1.508 (4)	N2—N3	1.294 (4)
C4—H4A	0.9700	N3—N4	1.355 (4)
C4—H4B	0.9700	O2—H2	0.8200
C5—C10	1.374 (4)	O1W—H1A	0.8904
C5—C6	1.385 (4)	O1W—H1B	0.9193
C6—C7	1.385 (4)		
			
N4—C1—N1	110.0 (3)	C5—C6—H6	119.9
N4—C1—H1	125.0	C7—C6—H6	119.9
N1—C1—H1	125.0	C8—C7—C6	120.6 (3)
O1—C2—O2	126.0 (2)	C8—C7—H7	119.7
O1—C2—C3	123.48 (19)	C6—C7—H7	119.7
O2—C2—C3	110.55 (18)	C9—C8—C7	119.5 (3)
N1—C3—C2	109.64 (18)	C9—C8—H8	120.3
N1—C3—C4	111.7 (2)	C7—C8—H8	120.3
C2—C3—C4	113.2 (2)	C8—C9—C10	120.4 (3)
N1—C3—H3	107.3	C8—C9—H9	119.8
C2—C3—H3	107.3	C10—C9—H9	119.8
C4—C3—H3	107.3	C5—C10—C9	121.1 (3)
C5—C4—C3	113.1 (2)	C5—C10—H10	119.4
C5—C4—H4A	109.0	C9—C10—H10	119.4
C3—C4—H4A	109.0	C1—N1—N2	108.12 (19)
C5—C4—H4B	109.0	C1—N1—C3	130.9 (2)
C3—C4—H4B	109.0	N2—N1—C3	121.0 (2)
H4A—C4—H4B	107.8	N3—N2—N1	106.1 (2)
C10—C5—C6	118.2 (3)	N2—N3—N4	110.8 (3)
C10—C5—C4	121.2 (3)	C1—N4—N3	105.0 (3)
C6—C5—C4	120.5 (3)	C2—O2—H2	109.5
C5—C6—C7	120.1 (3)	H1A—O1W—H1B	95.0
			
O1—C2—C3—N1	−7.0 (4)	C4—C5—C10—C9	179.6 (3)
O2—C2—C3—N1	174.4 (2)	C8—C9—C10—C5	0.3 (5)
O1—C2—C3—C4	−132.5 (3)	N4—C1—N1—N2	0.8 (3)
O2—C2—C3—C4	48.9 (3)	N4—C1—N1—C3	179.4 (2)
N1—C3—C4—C5	58.4 (3)	C2—C3—N1—C1	−68.8 (3)
C2—C3—C4—C5	−177.2 (2)	C4—C3—N1—C1	57.5 (3)
C3—C4—C5—C10	−106.6 (3)	C2—C3—N1—N2	109.7 (3)
C3—C4—C5—C6	73.5 (3)	C4—C3—N1—N2	−124.0 (3)
C10—C5—C6—C7	0.3 (5)	C1—N1—N2—N3	−0.4 (3)
C4—C5—C6—C7	−179.9 (3)	C3—N1—N2—N3	−179.2 (2)
C5—C6—C7—C8	0.3 (5)	N1—N2—N3—N4	0.0 (3)
C6—C7—C8—C9	−0.6 (6)	N1—C1—N4—N3	−0.8 (4)
C7—C8—C9—C10	0.3 (6)	N2—N3—N4—C1	0.5 (4)
C6—C5—C10—C9	−0.6 (5)		

### Hydrogen-bond geometry (Å, °)

**Table:**

*D*—H···*A*	*D*—H	H···*A*	*D*···*A*	*D*—H···*A*
O2—H2···O1W^i^	0.82	1.74	2.552 (2)	174
O1W—H1B···N4^ii^	0.92	1.98	2.903 (4)	177
O1W—H1A···N3^iii^	0.89	2.12	3.003 (3)	171

Symmetry codes: (i) *x*, *y*+1, *z*; (ii) −*x*+1/2, *y*, *z*+1/2; (iii) −*x*+1/2, *y*, *z*−1/2.
